# Scrub Typhus in Pregnancy: A Retrospective Observational Study From Primary Hospital of Southwest China

**DOI:** 10.1155/jotm/4922171

**Published:** 2026-10-11

**Authors:** Mengji Zhang, Su Pan, Xi Lan, Yueming Li, Xiaomei Wu

**Affiliations:** ^1^ The First People’s Hospital of Yunnan Province Kunming Yunnan China ypfph.com; ^2^ The Affiliated Hospital of Kunming University of Science and Technology Kunming Yunnan China kmust.edu.cn; ^3^ The People’s Hospital of Ximeng Va Autonomous County Pu’er Yunnan China

**Keywords:** azithromycin, eschar, fever, scrub typhus in pregnancy

## Abstract

**Background:**

Scrub typhus is an acute zoonotic disease caused by *Orientia tsutsugamushi*. There are limited reports on scrub typhus in pregnancy, and the misdiagnosis rate is relatively high, potentially leading to adverse maternal and fetal outcomes.

**Methods:**

This retrospective observational study analyzed 19 pregnant patients with scrub typhus admitted to the People’s Hospital of Ximeng Wa Autonomous County, Southwest China, from August 2023 to November 2025. Epidemiological, clinical, test, treatment, and outcome data were collected and descriptively analyzed.

**Results:**

All patients were farmers, with 73.68% reporting a history of outdoor activities. Common symptoms included fever (100%), myalgia (78.94%), headache (57.89%), and eschar (94.74%). Laboratory findings revealed elevated transaminases (ALT or AST 94.74%) and decreased eosinophil percentages (89.47%). Initial misdiagnosis occurred in 10.53% (upper respiratory infection). Among the 18 patients treated with azithromycin, fever resolution occurred within 1 to 5 days. No maternal deaths occurred. However, adverse fetal outcomes included spontaneous miscarriage (*n* = 1), embryonic arrest (*n* = 1), and embryo demise (*n* = 3). Among 19 cases, 14 resulted in live births; 2 premature infants required pediatric care, and all live‐born newborns showed normal development at six‐month follow‐up.

**Conclusions:**

Scrub typhus in pregnancy poses serious maternal‐fetal risks. Early clinical recognition and prompt azithromycin therapy are crucial for improving maternal and perinatal outcomes, especially in resource‐limited settings.

## 1. Introduction

Scrub typhus, caused by *Orientia tsutsugamushi* and transmitted by trombiculid mites, is an acute zoonotic disease prevalent in southern China during summer and autumn. Clinically, it is characterized by fever, headache, general body aches, eschar, and swollen lymph nodes. In endemic areas, scrub typhus may occur simultaneously with malaria, dengue, leptospirosis, or typhoid, etc. [1], leading to difficulties in early diagnosis. Failure to promptly diagnose patients with prolonged illness exceeding two weeks elevates the risk of multiple organ dysfunction, potentially leading to life‐threatening consequences. Reports on pregnancy complicated by scrub typhus are scarce both domestically and internationally, and the rate of misdiagnosis remains high. This condition can lead to stillbirth, miscarriage, premature birth, and even maternal mortality. The clinical data of 19 patients with scrub typhus in pregnancy, admitted to the People’s Hospital of Ximeng Wa Autonomous County in southwest China, are analyzed retrospectively as follows. The women in this area have a weak awareness of women’s health care and often fail to have regular prenatal check‐ups during pregnancy.

## 2. Materials and Methods

### 2.1. General Information

Nineteen patients diagnosed with scrub typhus in pregnancy were admitted to the People’s Hospital of Ximeng Wa Autonomous County between August 2023 and November 2025. The average age was (28.9 ± 6.5) years, and the median gestational week was 22 (ranging from 6 to 38 weeks).

### 2.2. Research Methods

Through the retrospective ICD code search for cases in the obstetrics and gynecology department of the hospital that were diagnosed with “scrub typhus” or “Orientia tsutsugamushi infection” as discharge diagnoses, the above 19 cases were obtained. Their epidemiological data, characteristic symptoms and signs, laboratory tests, and auxiliary examinations within 24 h of admission, as well as treatment and prognosis were analyzed.

### 2.3. Statistical Tools

Data were entered using Excel 2010 software, and relevant databases were created for statistical analysis. Quantitative data exhibiting a normal distribution were expressed as (*x* ± *s*), whereas count data were represented as percentages. Descriptive statistical analysis was performed on the observed indicators.

### 2.4. Diagnostic Criteria

Diagnostic criteria are based on the technical guidelines for the prevention and control of scrub typhus issued by the Chinese Center for Disease Control and Prevention [2].

#### 2.4.1. Possible Case

The inclusion criteria were as follows:

Criterion 1: Having epidemiological exposure history (e.g., exposure to endemic areas, sitting/lying on grass, etc.) AND fever, AND at least one of the following signs (lymphadenectasis or rash), with other diseases that could cause similar manifestations definitively excluded.

Criterion 2: In the absence of a clear epidemiological exposure history, simultaneously presenting with all three of the following signs during the epidemic season: fever, lymphadenectasis, and rash.

#### 2.4.2. Clinically Diagnosed Case

The inclusion criteria were as follows:

• Criterion 1: Meeting the criteria for a Possible case (as defined in Section 2.4.1) AND presenting with a characteristic eschar (a highly specific sign for scrub typhus)
• Criterion 2: Simultaneously presenting with all three of the following: epidemiological exposure history AND fever AND eschar.

### 2.5. IRB Ethical Approval and Patient Consent

This study was approved by the Ethics Committee of Ximeng County People’s Hospital (approval number: xmxyy2026012). All the patients submitted written informed consent.

## 3. Results

### 3.1. Epidemiologic Data

All 19 patients were from rural areas and their occupation was farming (100%). Fourteen patients (73.68%) had a clear history of working or resting in the grasslands before the onset of the disease, while the other 5 cases were unclear. The onset time showed a distinct seasonal pattern, concentrated in the period from June to November.

### 3.2. Clinical Data (Table 1)

All 19 cases (100.00%) had fever. The average duration of fever before admission was 5 (2–7) days. Among them, 12 cases (63.16%) had a maximum body temperature exceeding 39.1°C, 11 cases (57.89%) experienced headache, 15 cases (78.94%) experienced muscle pain, 11 cases (57.89%) had cough, and 5 cases (26.32%) had sore throat. Some also had anorexia (3 cases), shortness of breath (1 case), chest tightness (1 case), and vaginal bleeding (1 case). In all, 18 cases (94.74%) had eschars, and most patients had one eschar. Two cases found two eschars. Eschars were distributed not only in the concealed and smelly areas but also on the trunk and limbs. Four cases (21.05%) had superficial lymph node enlargement near the eschars. Two cases (10.53%) developed rashes. Five cases (26.32%) experienced edema.

**TABLE 1:** Basic information and clinical manifestations of the cases.

Case number	Age (years)	Gestational weeks	Gravidity parity	Medical history	Comorbidities	Fever	Days with fever before admission	Maximum body temperature (°C)	Head‐ache	Muscle pain	Cough	Sore throat	Eschar location	Lymphad‐enectasis	Rashes	Edema
1	36	16	G4P2	—	Hyperlipemia	+	2	39.4	+	—	—	—	Neck	+	—	—
2	15	33	G1P0	—	Hyperuricemia	+	3	39.2	+	+	—	—	Groin × 2	+	—	—
3	22	6	G2P1	—	Ovarian cyst	+	6	39.7	+	+	—	—	Elbow	—	—	—
4	30	29	G4P3	—	—	+	5	37.9	—	+	+	—	Hip, Elbow	—	—	+
5	31	31	G4P1	alcohol abuse	*Trichomonas* vaginitis	+	4	38.3	—	+	—	—	Thigh	—	—	—
6	22	38	G2P1	—	Oligohydramnios	+	5	39.5	—	+	+	—	Perineum	—	—	+
7^a^	28	12	G6P2	—	Urinary tract infection	+	6	40.3	—	—	+	+	—	—	—	+
8	33	15	G3P1	—	—	+	4	39.9	—	+	—	+	Loin	—	—	—
9	31	22	G5P2	—	—	+	5	39.5	+	—	+	+	Arm	—	—	—
10	31	15	G4P3	HBV carrier	—	+	7	38.1	+	+	+	—	Thigh	—	—	—
11	36	34	G4P2	—	Hyperlipemia	+	5	39.8	+	+	—	—	Thigh	—	—	—
12	30	24	G4P2	—	—	+	4	39.7	—	+	—	—	Mid‐abdomen	—	—	—
13^b^	31	35	G4P2	—	—	+	3	39.3	+	+	+	—	Loin	—	+	+
14	34	13	G5P2	—	—	+	6	39.3	+	+	+	—	Perineum	—	—	—
15	22	17	G3P1	—	—	+	5	38.9	+	—	+	+	Groin	+	+	—
16	40	17	G3P2	bronchial asthma	—	+	4	38.8	+	+	+	—	Breast	+	—	—
17	35	35	G4P2	—	Hyperlipemia	+	2	37.8	—	+	—	—	Back	—	—	—
18	23	32	G1P0	—	—	+	6	38.5	—	+	+	+	Chest	—	—	+
19	19	7	G1P0	—	Threatened abortion	+	7	39.5	+	+	+	—	Loin	—	—	—

^a^This case is the misdiagnosed case B.

^b^This case is the misdiagnosed case A.

### 3.3. Laboratory and Auxiliary Examinations (Table 2)

**TABLE 2:** Laboratory and auxiliary examinations of the cases.

Case number	WBC (× 10^9^/L)	NEUT (%)	LYM (%)	EOS (%)	HGB (g/L)	PLT (× 10^9^/L)	ALT/AST (U/L)	ALB (g/L)	TBIL (µmol/L)	CRE (µmol/L)	BUN (mmol/L)	Weil–Felix test	Widal test	Malaria smear
1	11.43	77.4	16.4	0.2	129	179	37/91	41.8	15.4	49	1.57	—	—	—
2	5.68	74	18.9	0.2	120	179	39.1/69.5	36.8	8.5	56	2.37	—	—	—
3	7.69	79.5	11.2	0.3	137	86	169.1/194.6	32.7	8.1	68	3.39	—	—	—
4	9.33	56.9	31.2	0.3	107	158	58/82	26.7	22.2	40	1.86	—	—	—
5	10.04	80.6	13.9	0.3	130	102	13.4/57.9	38.6	27.5	55.1	2.32	—	—	—
6	10.91	82	7.3	0.2	99	53	38.3/92.3	31	20.1	51.3	1.9	—	—	—
7^a^	5.58	66.3	24.3	0.3	132	168	41/74.5	33.4	6.1	52	1.14	—	—	—
8	2.48	68.5	27.3	0.2	114	166	53/164	37.8	6.5	45	2.16	—	—	—
9	7.01	78	11.4	0.5	111	71	88/167	33.6	23.3	80	4.99	—	—	—
10	5.98	73.1	18.9	0.2	135	89	57/147	41.8	17.1	52	3	—	—	—
11	6.62	74.9	15.8	0.2	91	102	54/138	35.5	16.7	44	1.15	—	—	—
12	5.18	84.8	11.5	0.4	125	98	62/88	35.2	6.4	41	1.38	—	—	—
13^b^	9.61	81.7	10.1	0.4	118	133	22/50	30.8	17.6	59	2.2	—	—	—
14	3.05	77	16.8	0.3	143	109	53.3/93.6	34.9	7.7	39	1.63	—	—	—
15	9.65	63.5	28	0.4	111	171	48.2/44.9	34.9	9.2	45	1.69	—	—	—
16	8.49	77.6	10.7	0.5	126	153	61/68	30.8	8.7	43	2.17	—	—	—
17	5.24	76.9	13.7	0.3	130	150	16.3/15.9	34.8	4.7	53.8	1.78	—	—	—
18	6.16	70.3	18	0.2	102	103	1624.7/100.5	30.3	26.1	53.1	2.29	—	—	—
19	11.76	71.8	17.3	0.3	131	35	92/137	35.7	14.9	71	3.69	—	—	—

^a^This case is the misdiagnosed case B.

^b^This case is the misdiagnosed case A.

#### 3.3.1. Blood Routine

The total number of white blood cells (WBCs) was within the normal range in 13 cases (68.42%), increased [(10.04–11.76) × 10^9^/L] in 4 cases (21.05%) and decreased [(2.48–3.05) × 10^9^/L] in 2 cases (10.53%). The percentage of neutrophils (NEUT) was increased in 15 cases (78.95%) but remained normal in 4 cases (21.05%). The percentage of lymphocytes (LYM) was decreased in 15 cases (78.95%) but remained normal in 4 cases (21.05%). The percentage of eosinophils (EOS) was decreased in 17 cases (89.47%) but remained normal in 2 cases (10.53%). The hemoglobin (HGB) level was normal in 17 cases (89.47%), and mild anemia (91–99 g/L) was present in 2 cases (10.53%). The platelet (PLT) count was normal in 13 cases (68.42%) but decreased in 6 cases [(35–98) × 10^9^/L].

#### 3.3.2. Blood Biochemistry

The liver function tests showed that the total bilirubin (TBIL) levels were normal in all 19 cases. The alanine aminotransferase (ALT) levels were elevated (37–1627.4 U/L) in 16 cases (84.21%), and the aspartate aminotransferase (AST) levels were elevated (50–194.6 U/L) in 17 cases (89.45%). The albumin (ALB) levels were decreased (26.7–34.9 g/L) in 11 cases (57.89%). The renal function tests showed that the creatinine (CRE) levels were normal in all 19 cases, and the blood urea nitrogen (BUN) levels were decreased (1.14–2.17 mmol/L) in 11 cases (57.89%).

#### 3.3.3. Immunologic Test

Weil–Felix tests were conducted on 11 cases, and the results were all negative (OXK titer < 1:160). Widal tests were performed in 11 cases and malaria blood smear in 4 cases; all results were negative.

#### 3.3.4. Ultrasonography

None of the 19 cases showed any signs of liver or spleen enlargement. One case (at 7 weeks of pregnancy) was indicated as having uterine cavity effusion.

### 3.4. Diagnostic Assessment

According to the diagnostic criteria, 18 cases were clinically diagnosed, and 1 was classified as a possible case. At the initial diagnosis, 2 cases (10.53%) were misdiagnosed as upper respiratory tract infections.

Case A was rectified after a thorough physical examination on the third day of admission, while Case B, lacking eschars throughout the illness, was initiated with azithromycin diagnostic treatment on the fifth day of admission, based on the clinical suspicion of scrub typhus.

### 3.5. Treatment and Outcome

#### 3.5.1. General Treatment

Patients were advised to rest in bed. Supportive care included liver protection, gastric mucosal protection, myocardial nutrition, correction of electrolyte imbalances, nutritional support, and oxygen therapy. For patients in the second or third trimester of pregnancy, fetal heart rate and fetal movements were closely monitored. In cases of high fever, physical cooling was administered.

#### 3.5.2. Etiologic Treatment

In the 17 cases that were clinically diagnosed, azithromycin was administered intravenously at a dosage of 0.5 g per day for a duration of 7 to 10 days. The duration of azithromycin treatment was based on guidelines and physician discretion, guided by clinical response. Most patients received 7 days of treatment, with extension to 10 days if fever persisted beyond 72 h. Three patients were slow to defervesce and received 10 days of treatment. In case A, after 3 days of treatment with cefoperazone sulbactam, the diagnosis was corrected and treatment was changed to azithromycin. On the 4th day, the patient developed a non‐raising body temperature and sitting‐up breathing. It was considered as septic shock and heart failure. The patient was transferred to a higher‐level hospital for treatment. Doxycycline was administered intravenously at a dose of 0.2 g per day for 7 days. In case B, after 4 days of treatment with cefazolin sodium and cefoperazone sulbactam, the medication regimen was adjusted on the 5th day, and the treatment was changed to intravenous administration of azithromycin at a dose of 0.5 g per day for 7 days.

#### 3.5.3. Outcome (Table 3)

The body temperature of 18 patients returned to normal within 1 to 5 days after receiving azithromycin treatment. Among the 4 patients in the first trimester, 1 case (7 weeks of gestation) experienced a spontaneous miscarriage during hospitalization. Another patient (6 weeks of gestation) underwent a B‐ultrasound examination 1 week post‐discharge, which indicated embryonic arrest. The remaining 2 cases continued their pregnancies until full‐term delivery. Seven patients in the second trimester maintained their pregnancies after discharge from the hospital. Among these, 1 patient (17 weeks of gestation) experienced embryo demise at 28 weeks of gestation. Of the remaining 6 patients, 4 achieved full‐term natural deliveries, while 2 underwent full‐term cesarean sections. In the third trimester, 8 patients were observed, 2 of whom (at 29 weeks and 32 weeks of gestation, respectively) experienced embryo demise at 38 weeks and 36 weeks of gestation, respectively. Case A underwent a cesarean section at 36 + 3 weeks of gestation in a higher‐level hospital. Among the remaining 5 patients, 3 had full‐term natural deliveries, 1 had a full‐term cesarean section, and 1 had a premature natural delivery. All the patients recovered from the hospital, with no deaths reported. Of the 14 live‐born newborns, 2 were transferred to the pediatric department due to premature birth and subsequently discharged, while the remaining 12 were discharged with their mothers. A follow‐up conducted six months later via telephone revealed that all live newborns experienced no feeding difficulties, did not develop any serious conditions necessitating hospitalization, and exhibited generally normal cognitive and motor development.

**TABLE 3:** Mode of delivery and fetal outcomes of the cases.

Case Number	Gestational weeks of delivery or miscarriage	Mode of delivery	Fetal outcome	Interval weeks between hospital Discharge and confirmed fetal death
1	38	Cesarean section	Live birth	—
2	35	Vaginal delivery	Live birth	—
3	7	—	Miscarriage	—
4	38	Vaginal delivery	Intrauterine death	9
5	37	Vaginal delivery	Live birth	—
6	38	Cesarean section	Live birth	—
7^a^	40	Vaginal delivery	Live birth	—
8	39	Vaginal delivery	Live birth	—
9	40	Vaginal delivery	Live birth	—
10	40	Vaginal delivery	Live birth	—
11	40	Vaginal delivery	Live birth	—
12	40	Cesarean section	Live birth	—
13^b^	36	Cesarean section	Live birth	—
14	38	Cesarean section	Live birth	/
15	40	Vaginal delivery	Live birth	—
16	28	Vaginal delivery	Intrauterine death	10
17	40	Vaginal delivery	Live birth	—
18	36	Vaginal delivery	Intrauterine death	3
19	7	—	Miscarriage	—

^a^This case is the misdiagnosed case B.

^b^This case is the misdiagnosed case A.

The trimester‐stratified summary presenting the number of patients, key clinical features, and pregnancy outcomes is shown in Table 4.

**TABLE 4:** Trimester‐stratified summary table.

Trimesters	Number of patients (*n*)	Fever [*n* (%)]	Eschar [*n* (%)]	WBC normal/Decreased [*n* (%)]	EOS% decreased [*n* (%)]	ALT/AST elevated [*n* (%)]	Recovered patients [*n* (%)]	Adverse fetal outcomes [*n* (%)]
First trimester	4	4 (100)	3 (75)	3 (75)	4 (100)	4 (100)	4 (100)	2 (50)
Second trimester	7	7 (100)	7 (100)	6 (85.71)	5 (71.43)	7 (100)	7 (100)	1 (14.29)
Third trimester	8	8 (100)	8 (100)	6 (75)	8 (100)	7 (87.5)	8 (100)	2 (25)
Total	19	19 (100)	18 (94.74)	15 (78.95)	17 (89.47)	18 (94.74)	19 (100)	5 (26.32)

## 4. Discussion

An epidemiological study conducted in Dehong Dai and Jingpo Autonomous Prefecture, Yunnan Province, China from 2010 to 2024 revealed an annual average reported incidence rate of scrub typhus of 71.88 per 100,000 people, with a year‐on‐year increase [3]. Another large‐scale population cohort study in southern India revealed that the clinical infection rate of scrub typhus was as high as 6.0 per 1000 people, and approximately 21.6% of the cases required hospitalization [4]. In areas where scrub typhus is endemic, such as in southwestern China, India, and Southeast Asia, reports of scrub typhus cases during pregnancy are gradually increasing. The clinical manifestations of pregnant women are similar to those of nonpregnant individuals. The typical symptoms include fever, headache, myalgia, cough, and gastrointestinal discomfort. The characteristic eschar serves as a crucial diagnostic indicator although its prevalence varies significantly across studies, ranging from 33% to 92.5% [5–8]. The variability in eschar occurrence may be attributed to the differing eschar formation capabilities of various strains of *O. tsutsugamushi* across geographical regions [9]. In addition, the host’s immune response, the timing of the examination, and skin pigmentation may also affect the detection of the eschar [10]. Patients lacking an eschar pose a greater challenge for diagnosis. Among the 19 cases in this study, 18 exhibited one or two eschars. No instances of eschar were identified as being susceptible to early misdiagnosis.

Scrub typhus in pregnancy may result in various complications, ranging from mild to life‐threatening. Following infection, pregnant women face an elevated risk of experiencing severe complications such as toxic hepatitis, acute kidney injury, pneumonia, encephalitis, heart failure, acute respiratory distress syndrome, nervous system involvement, hearing impairment, and multiple organ dysfunction [1, 6, 11–14]. As a result, the condition poses significant risks, increase in treatment costs, and a grim prognosis. In this study, the incidence of hepatic injury reached 94.74%, with no reported cases of elevated bilirubin. This absence may be attributed to the brief duration of the disease during treatment, all less than 2 weeks. Severe hepatic injury can advance to acute liver failure, a significant contributor to maternal mortality [15]. Case A in this study exhibited multiple organ dysfunction and critical condition; however, none of the 19 cases led to maternal mortality, aligning with the low maternal mortality rate (0%–3%) reported in various studies when timely diagnosed and appropriately managed [6, 16–18].

However, scrub typhus in pregnancy poses a particularly prominent threat to the fetus. Although the overall incidence of adverse fetal outcomes varies among different studies, the risk of spontaneous abortion is extremely high when infected in the early stage of pregnancy. In this study, we report a case of early pregnancy infection characterized by moderate thrombocytopenia, which subsequently resulted in miscarriage. Previous research has indicated that thrombocytopenia serves as a significant marker of systemic vasculitis and endothelial damage in scrub typhus [19]. This systemic vascular injury not only impacts maternal organs but may also lead to adverse fetal outcomes by affecting placental blood perfusion. A large‐scale study in Southwest China shows that the miscarriage rate after infection in the early stage of pregnancy is as high as 64.5%, and the decreased platelets were significantly associated with adverse fetal outcomes in the univariate analysis [6]. The occurrence of intrauterine fetal death, stillbirth, and preterm delivery during the middle and late stages of pregnancy in various domestic and international studies is around 50% [6, 16]. In this study, one case of infection occurred during the late stage of pregnancy, characterized by severe liver function impairment and ALT > 1000 U/L. Following treatment, the ALT level remained abnormal, and embryo demise was reported 3 weeks post‐discharge. Impaired liver function can result in coagulation disorders. In this case, although the patient’s coagulation function normalized upon discharge, the abnormal liver function might adversely affect the metabolism of carbohydrates, lipids, and proteins. This disruption might have interfered with the supply of essential nutrients necessary for normal fetal development, ultimately leading to unfavorable fetal outcomes. In a cause‐and‐death surveillance study in Bangladesh, *O. tsutsugamushi* DNA was detected in autopsy specimens after stillbirth and neonatal death, directly confirming its correlation with perinatal mortality [20]. In this study, among the four patients with first trimester infection, two had a miscarriage. Among the 15 cases in the second and third trimester, three had embryo demise and two had premature birth. The economy in this region is relatively underdeveloped, and women generally have low educational attainment. Post‐discharge, routine ultrasound scans and monitoring of fetal activity for pregnant women are frequently neglected. The elevated risk of unfavorable pregnancy outcomes maybe associated with irregular prenatal check‐ups, yet the potential contribution of vertical transmission of scrub typhus cannot be discounted.

In epidemic regions, pregnant women with unexplained fever should be assessed for scrub typhus. Diagnosis relies on clinical suspicion and is typically confirmed by serological tests (e.g., Weil–Felix test, immunofluorescence test, IgM enzyme‐linked immunosorbent assay) or pathogen nucleic acid tests. The Weil–Felix test has been conducted in numerous primary hospitals; however, the positive rate remains relatively low, and both sensitivity and specificity are suboptimal. Additionally, the test’s performance is time‐dependent, which increases the risk of false‐negative results. In this study, 11 patients who underwent the Weil–Felix test returned negative results. The People’s Hospital of Ximeng Va Autonomous County is currently unable to carry out IFA, IgM enzyme‐linked immunosorbent assay (ELISA), or *O. tsutsugamushi* PCR test. Case B highlights the possibility of misdiagnosis under such laboratory testing limitations. Although the samples could be referred to the laboratories of higher‐level hospitals for testing, due to the drawbacks such as the cost being borne by the patients themselves and the 2 to 3 days required for external testing, none of the 19 patients, along with their newborns or fetuses, underwent the abovementioned tests.

It is worth noting that 89.47% of the cases in this study showed a decrease in the percentage of EOS, suggesting that in the epidemic areas, a decrease or even disappearance of the percentage of EOS in patients with fever of unknown cause should raise suspicion of scrub typhus. Meanwhile, it is still necessary to combine detailed history collection and physical examination, and carry out the laboratory tests for scrub typhus as soon as possible. A study by Dr. He et al. on the clinical characteristics and related risk factors of severe scrub typhus also revealed that patients with scrub typhus generally have a decreased or absent percentage of EOS [21].

Sil A et al. pointed out in their commentary that monotherapy is recommended in the treatment of scrub typhus [22]. Azithromycin is the preferred drug for treating scrub typhus in pregnancy. Multiple studies have confirmed that the treatment with azithromycin (500 mg once or twice a day for a course of 5 days) is effective and safe for the fetus and can lead to a good pregnancy outcome [7, 19, 23]. In severe scrub typhus in pregnancy, saving the mother’s life and improving the prognosis of the fetus should be the primary principles. The benefits of doxycycline treatment are clear [24].

This retrospective single‐center study was conducted in the primary hospital in Southwest China where medical resources are limited. The absence of IgM ELISA or PCR for scrub typhus might impede diagnostic accuracy. Similarly, due to the absence of laboratory confirmation, it was impossible to completely rule out other endemic febrile diseases, and there was also a lack of assessment of vertical transmission to the fetus, placenta, or newborn. Moreover, the relatively limited sample size might compromise statistical power and result extrapolation. Following data collection, we recommended enhancements in scrub typhus laboratory testing to the local medical administrative department. In the future, it will be necessary to conduct scrub typhus related tests on live newborns, fetuses or placental tissues with adverse pregnancy outcomes, and extend the follow‐up to assess the vertical transmission from mother to child.

## 5. Conclusion

Scrub typhus in pregnancy is an infectious disease with potential fatal risks, which may lead to a high proportion of miscarriages, stillbirths and premature births. The clinical presentation of this disease is varied and sometimes unusual. In endemic regions, it should be routinely considered in the differential diagnosis of fever of unknown origin. Early clinical recognition and the prompt initiation of azithromycin treatment are essential for improving maternal and infant outcomes. In primary hospitals lacking IgM ELISA or PCR tests for scrub typhus, detailed history collection and physical examination, as well as finding indicators from common hematological tests that can assist in the diagnosis of scrub typhus, are of great significance for improving the prognosis of mothers and infants in resource‐limited areas. Azithromycin is the preferred drug for treating scrub typhus in pregnancy, but in severe scrub typhus, the benefits of doxycycline treatment are clear. Including the comparator group to assess the diagnostic utility of eosinopenia, further clarifying the optimal treatment plan, and enhancing the understanding and management level of vertical transmission and congenital infections are the future research directions.

## Author Contributions

Conceptualization, Mengji Zhang and Xiaomei Wu; methodology, Mengji Zhang; formal analysis, Su Pan; investigation, Mengji Zhang and Su Pan; resources, Xi Lan; data curation, Yueming Li; writing–original draft preparation, Mengji Zhang; writing–review and editing, Xi Lan; visualization, Mengji Zhang; supervision, Xiaomei Wu; project administration, Xiaomei Wu; funding acquisition, Xiaomei Wu.

## Funding

This study was supported by Central Government Guidance Fund for Local Science and Technology Development (grant number 202407AB110013).

## Disclosure

All authors have read and agreed to the published version of the manuscript.

## Conflicts of Interest

The authors declare no conflicts of interest.

## Supporting Information

Additional supporting information can be found online in the Supporting Information section.

## Supporting information


**Supporting Information** CARE‐checklist 0616.

## Data Availability

The data that support the findings of this study are available from the corresponding author upon reasonable request.
