# Supplementary material for: Scrub Typhus in Pregnancy: A Retrospective Observational Study From Primary Hospital of Southwest China
Source: J Trop Med. 2026 Oct 11;2026:4922171. doi: 10.1155/jotm/4922171 (PMC13655380; doi:10.1155/jotm/4922171)
Supplement: Supplementary file 1 [file JOTM-2026-4922171-s001.pdf]

CARE Checklist of information to include when writing a case report

CARE case report guidelines

(cc) BY-NC-ND

Topic	Item	Checklist item description	Reported on Line
Title	1	The diagnosis or intervention of primary focus followed by the words “case report”	Line 1-2 / Page 2
Key Words	2	2 to 5 key words that identify diagnoses or interventions in this case report, including "case report"	Line 24-25 / Page 2
Abstract (no references)	3a	Introduction: What is unique about this case and what does it add to the scientific literature?	Line 6-8 / Page 2
	3b	Main symptoms and/or important clinical findings	Line 11-14 / Page 2
	3c	The main diagnoses, therapeutic interventions, and outcomes	Line 14-19 / Page 2
	3d	Conclusion—What is the main “take-away” lesson(s) from this case?	Line 20-22 / Page 2
Introduction	4	One or two paragraphs summarizing why this case is unique (**may include references**)	Line 34-39 / Page 2
Patient Information	5a	De-identified patient specific information	Line 24-27 / Page 3
	5b	Primary concerns and symptoms of the patient	Page 3 /Tab.1
	5c	Medical, family, and psycho-social history including relevant genetic information	Page 3 /Tab.1
	5d	Relevant past interventions with outcomes	Page 3 /Tab.1
Clinical Findings	6	Describe significant physical examination (PE) and important clinical findings	Page 4 /Tab.2
Timeline	7	Historical and current information from this episode of care organized as a timeline	Not Applicable
Diagnostic Assessment	8a	Diagnostic testing (such as PE, laboratory testing, imaging, surveys)	Line 26-27 / Page 4
	8b	Diagnostic challenges (such as access to testing, financial, or cultural)	Line 25-30 / Page 7
	8c	Diagnosis (including other diagnoses considered)	Line 1-3 / Page 5
	8d	Prognosis (such as staging in oncology) where applicable	Not Applicable
Therapeutic Intervention	9a	Types of therapeutic intervention (such as pharmacologic, surgical, preventive, self-care)	Line 9-23 / Page 5
	9b	Administration of therapeutic intervention (such as dosage, strength, duration)	Line 15-16 / Page 5
	9c	Changes in therapeutic intervention (with rationale)	Line 16-23 / Page 5
Follow-up and Outcomes	10a	Clinician and patient-assessed outcomes (if available)	Page 5 /Tab.3
	10b	Important follow-up diagnostic and other test results	Page 5 /Tab.3
	10c	Intervention adherence and tolerability (How was this assessed?)	Line 11 / Page 6
	10d	Adverse and unanticipated events	Page 5 /Tab.3
Discussion	11a	A scientific discussion of the strengths AND limitations associated with this case report	Line 2-9 / Page 8
	11b	Discussion of the relevant medical literature **with references**	L 16 / P 6--L 1 /P 8
	11c	The scientific rationale for any conclusions (including assessment of possible causes)	L 16 / P 6--L 1 /P 8
	11d	The primary “take-away” lessons of this case report (without references) in a one paragraph conclusion	Line 12-21 / Page 8
Patient Perspective	12	The patient should share their perspective in one to two paragraphs on the treatment(s) they received	Not Applicable
Informed Consent	13	Did the patient give informed consent? Please provide if requested	Yes ☑ No ☐
